# I am only a nurse: a biographical narrative study of a nurse’s self-understanding and its implication for practice

**DOI:** 10.1186/s12912-015-0073-y

**Published:** 2015-04-28

**Authors:** Ellen Ramvi

**Affiliations:** Department of Health Studies, The University of Stavanger, Faculty of Social Sciences, Stavanger, N-4036 Norway

**Keywords:** Learning from experience, Professional (nurse) development, Self-understanding, Biographical narrative interpretive method, Professional identity formation, Lived experience

## Abstract

**Background:**

The personal is a vital part of professional nursing practice. From a psycho-social perspective, nurses produce and reproduce conceptions of the Self through experience. A literature search on nurses’ self-understanding in a psycho-social perspective yields no results. Hence, the aim of this study was to investigate personal and professional experiences that may have formed the self-understanding of a nurse, and how this self-understanding may have influenced her professional practice.

**Methods:**

Using a single case approach, I conducted a Biographical Narrative Interview with a 50-year-old experienced Norwegian nurse. I asked the nurse to tell me the story of her life and how her work has affected her and possibly changed the way she saw herself. The overall aim of the interpretation was to understand the historically situated subjectivity in terms of the nurse’s personal, social and professional constraints and chosen options.

**Results:**

The nurse’s narrative of her life story made it possible to trace a common theme throughout her experiences, the experience of being “only a nurse”. The nurse experienced a low status, as well as a downgrade in the competence needed to deliver quality care in professional relationships. She felt it difficult to identify with the experience of being on the bottom of the social ladder and to identify with the female, mothering ideal connected to nursing. She desired a better position, and wanted to identify with strong women. In contrast to reality, her self-understanding influenced her relationship with her patients, her professional pride and her further professional development.

**Conclusions:**

This study shows that the professional practice of a nurse was informed by her self-understanding. This study suggests that the individual nurse must be given the opportunity to explore her professional vulnerability based on the assumption that it is both personally and socially constituted. This study indicates that the exploration of a nurse’s self-understanding is one way to contribute to professional development.

## Background

In an exploration of the nature of nursing and the function of the nurse within a 21^st^ century health-care system, researchers conclude that an understanding of nursing practice places demands not only on the technical competence of the nurse, but also on “the personhood” of the nurse [1]. The nurse has to be personally involved and “use herself” [2-4], and it is difficult to say where the personal ends and the professional begins. The assumption that the personal informs the professional and vice versa is supported by several studies e.g. [2,5-8]. Iranmanesh *et al*. [8] address this by saying *“a particular nurse’s understanding of care and the content of that care depend on his or her life story*” (p. 208). Moreover, the assumption is that nurses’ professional identities develop throughout their lifetimes [9]. As Henderson [5] puts it: “…*the self which is private person and the self which is nurse are constantly interacting and changing one another*” (p. 135). A number of studies on nurses’ self-concept e.g. [9,10] have been published, as they are closely linked to-, yet separate from professional identity. Arbon [11] echoes the understanding of nursing practice as something one “is”. He says: “*The uniqueness apparent in the ways that individual nurses relate to others and enact their practice is interesting, and seems to be associated with personal understandings about who they are and what is important to them. These features of the individual appear, often, to be related to their life experience(s) and not just experience in clinical situations as a nurse; and this is reflected consistently in nurses’ stories about practice*” (p. 150).

The relationship between nurse and patient has historically evolved from a subject-object- to a subject-subject relationship, thereby leading to an intensified need for self-reflection and introspection by nurses and other relationship workers [12]. Thus, the importance of self-awareness [13] in the nurse-patient relationship is underscored.

As its point of departure, the present study makes an underlying assumption that nurses produce and reproduce conceptions of the self through experience. A psycho-social perspective was adopted which assumes that the psyche and one’s social life are mutually constitutive [14]. In light of this perspective, the experience of the self is a substantial dimension in our inner, subjective world that affects our attitudes and our choices, through which our relationships to other people are also affected. However, the experience of the self is only partially consciously available to the individual, as we often avoid engaging with painful matters through all sorts of means [14]. In this perspective, it is a great challenge to perceive a clear mental boundary set between not-I and I. It can be difficult to distinguish the thoughts I attach to another, but that are really my own, and the thoughts and emotions that belong to the other person. Using the “self” is thus to be able to possess self-knowledge, which is made difficult by projective processes. Hence, we are “defending subjects” [15].

As it appears here, the psycho-social perspective is closely related to psychoanalytic thinking. However, in a psycho-social perspective, the subject and the inner world cannot be understood without knowledge of the subjective experience in the world, and vice versa. The characteristic of the psycho-social perspective is therefore that attention is paid to the dialectic between inner experience and the social conditions of the experience [16]. A psycho-social approach is of value in the investigation of nurses’ life experiences, social relationships and beliefs, while also responding to individual needs, strengths and vulnerabilities that may be developmental or constitutional in origin. It is in this perspective that I use the word *self-understanding* in this study. It is not because it necessarily differs from other concepts mentioned above, but because what I am after is a self-understanding that also considered unconscious processes. A literature search on nurses and self-understanding in this psycho-social perspective yielded no results. Hence, the aim of this present study was to investigate personal and professional experiences that may have formed the self-understanding of a nurse, and how this self-understanding may have influenced her professional practice. Whereas standards seek to define and prescribe the professional role that nurses play, nurses’ self-understanding is complex and socially situated within lived experiences.

## Method

The psycho-social perspective in this study demands a methodology that avoids psychological reductionism on the one hand and an over-social view of the subject on the other. To help capture the nurse’s way of making sense of her personal, social and professional world as it evolved over time [17], the Biographic Narrative Interpretive Method (BNIM) [18,19] was chosen.

In this article, I present a single individual. In accordance with Creswell [20], the central purpose or focus of a narrative approach is on the life of an individual. Within psycho-social research, a priority is also given to a holistic analysis of an individual case [21,15]. The single case approach chosen in this article gives an opportunity to explore in detail one nurse’s situated subjectivity. This in-depth inquiry can lead to a basic understanding of how self-understanding (affected by conscious and unconscious experiences from personal and professional life) impacts on a nurse’s professional practice. I agree with Chamberlayne and King [17], who sum up the advantages of single case approach in this way: (i) It avoids the invocation of grand narratives; (ii) It retains the individuality of the case while highlighting its key dynamic; and (iii) It elucidates the process of analysis and interpretation. For this study, it is important how the method helps the informant to relate the personal to the social, and to probe unconscious, latent meanings. The study will therefore not be able to simply present the nurse’s account of her situation, but to also make an interpretation of underlying personal meaning. Chamberlayne and King [17] refer to a “double significance”; for while the meanings are highly specific to the individual self-understanding and professional identity of the nurse, they are also “cultural artefacts” (p. 98) that arise from a particular social and cultural context. It is not assumed the nurse will “tell it like it is” [15], but through analysis the story is seen to offer a means to understand the nurse as a situated subject [18]. Within a psychosocial approach, the task of theory moves from codifying abstract regularities and generalizing across cases, to making thick descriptions and generalizing within them [22].

### Sample

The study was approved by the Norwegian Social Science Data Service in Bergen, Norway (project no. 19246) and by the research unit at a university hospital in Norway. A purposive sample [23] of one registered nurse took part in the study. Head nurse from a surgical unit at a university hospital recruited an experienced nurse, and both written and oral information was provided. The nurse gave her written consent to the interview, and was informed about her right to full confidentiality and to be able to withdraw from the study at any time with no negative consequences [24]. In order to protect the identity of the nurse in the case presented, some sensitive details were omitted and some were also changed in the published story.

### Data collection

The BNIM is a structured and staged method of interviewing and case-based analysis. The narrative interview primarily involves two sub-sessions [18,19], with the first sub-session starting with a “single question aimed at inducing narrative” (SQUIN). The interviewer facilitates open responses, but never directs or comments. The interviewee then decides on the shape of her response, which ends the first sub-session. The SQUIN presented to the nurse was: *Can you tell me the story of your life and how your work has affected you and maybe changed the way you see yourself? Include those events and experiences that were personally important to you. Begin wherever you like and take as much time as you need.*

In the second sub-session, the interviewee was asked to only tell “more stories” and give more narration. She was pushed towards particular incident narratives (PINs) and away from theories, arguments or justifications. Moreover, she was asked about the items she raised, in the order she raised them and using her own phrases.

Maria is a 50-year-old nurse, whose interview was conducted at the hospital in a quiet room connected to the unit where she worked. At first, she told her life story (sub-session one) in 10 minutes (upbringing, schooling, travels, higher education, family activities). However, when I (the interviewer) was quiet, she continued her story with some breaks until she stopped the interview after 27 minutes. Following a short, 10-minute break, we began sub-session two, pushing for PINs. This part of the interview lasted 1.5 hours, with the interview lasting two hours. The presentation of her story includes stories from both sub-sessions and follows the order of events as she relayed them.

### Analysis

The BNIM analysis implies a thorough process leading towards an understanding of the individual case, with the process interpretation procedures explained in Wengraf [18,19].

The interview was taped and transcribed verbatim, and as I was transcribing I was engaging in a free associative process – a first reading to identify my initial response as a researcher [19]. I made use of a three-column form as I transcribed, in which the centre column was the transcription while the right column was continuously filled with my initial free associative responses to what I heard. In the left column, I recorded both the time and marks about text sorts (a manner of speaking such as report, argumentation, evaluation, PIN, etc.).

The BNIM has two basic interpretive tracks, which I first interpreted separately and then together: (i) the living of the lived life, and (ii) the telling of the told story. In addition to the analyses conducted by the researcher, the BNIM relied on the involvement of an interpretation panel in the beginning of the analysis process to help me (the researcher) expand my imaginative capacity and avoid being left alone with my inevitable sub-cultural spontaneous presuppositions, prejudices and blind spots. I arranged two panels, with each lasting three hours. I started the two tracks’ interpretation by creating the chronology of “objective” biographical data (the “living of the lived life”). The first panel session was interpreted on this chronological biographical data track, whereas the second panel session was interpreted on the track of “the telling of the told story”, i.e. the sequence of segments in which the telling of the told story was put forth. In these panels, we developed future-blind hypotheses and counter-hypotheses, using a chunk-by-chunk interpretation. In other words, the interpretive panel tries to simulate the interviewee’s own subjective living of life and telling of life as it is revealed to them text segment by text segment. By multiplying the different ways this particular life could have been lived (first panel) and this narrative told (second panel), I was attempting to get a grasp on the double experience of the nurse, both the “then” subjectivity and the “now” subjectivity (during the interview).

Through the use of panels, the BNIM takes seriously not only that experiences in life are personal, but also that the meaning of these experiences is socially and discursively appropriated, informed by cultural discourses and relative to the situation. The panel is supposed to be heterogeneous. However, the panel I used in this particular case had some key features in common with each other and with the informant, namely that they were women, and were within the same age group as the inclusion criteria for the nurses in the study. The reason for this choice was that this panel would be able to recognize and identify with the nurse’s story, the time she was brought up and the political and societal environment she grew up in. Two of the three panels’ members were also nurses in different positions – one as a research nurse in a competence centre and one as the head of an institute of health at a university. The third member of the panel had an education as a preschool teacher and worked as a lecturer at the preschool teacher education at a university. Nonetheless, despite the similarities between the panel members and the nurse, the three women also represented differences, as experiences are always subjective and every individual hold different outlooks and attitudes. The panel expanded the range of considered interpretations to the case material because they were able to identify and distance themselves, while feeling both familiar and strange in relation to the story of the nurse.

After this valuable input to my interpretation, I continued the twin-track interpretation work on my own, attempting to reconstruct (imagine) how the interviewee experienced the successive experiences and actions of her life, and, at different moments, how she made sense (or failed to make sense) of that evolving life. In particular, how did she make sense of that entire evolving life being studied by telling it in the specific way that she did? I summarized both “the living of the lived life” and “the telling of the told story” as according to my current understanding of the pattern of each track.

In addition, I conducted a microanalysis at the annual conference of the International Group for Psychosocietal Research, which is held in Dubrovnik, Croatia every year, and where I am a member. Here, an interpretation from the in-depth hermeneutic European critical theory tradition, known as “the interpretation group method” [25,26], was conducted. The method involved an in-depth reading of part (two pages) of the transcript in a group of six researchers, which included an interpretation of also being aware of our (the interpreters) own responses and other associations.

The last step in the interpretation process was to bring the two interpretive tracks and the microanalyses together in a “case history” that integrated everything. The overall task for the interpretation was to understand the historically situated subjectivity in terms of the nurse’s personal, social and professional constraints and chosen options, the understanding of which goes beyond the self-understanding of the biographer [27].

First, I present the case history (“The life story of Maria”), and then I subsequently present a psycho-social interpretation of her life story in the Discussion section of the article.

### Study limitations

This in-depth case study sought to understand the lived experience of one specific nurse.

Therefore, the quest was not for objectivity and explanation, but for subjective meanings and deeper understandings [18]. The BNIM helps to facilitate an understanding of both the “inner” and “outer” worlds of “historically-evolving persons-in-historically-evolving situations”, and particularly the interactivity of inner and outer world dynamics. Thus, an analysis of narratives provided by other nurses would necessarily bring different experiences and a variety of self-understandings, influencing professional practice differently, however, these are not within the scope of this particular study. Moreover, one of the principles in the BNIM is to elicit narratives in an uninterrupted way (sub-session one). The story told by the nurse sets a framework for what I come to know (in sub-session two), as I am restricted to asking questions within this framework put up by the nurse in her first story. Finally, I stated earlier in this article that I encountered the “defended subject” (the nurse) in my theoretical perspective. A consequence of this stand is the assumption that I am the “defended researcher”. Thus, any interpretation of the nurse’s story is incomplete, unfinished and a rendering from my own perspective as a female nurse and researcher, born within the same period of time as the nurse. The use of panels and peer groups in data analyses is a way to meet this challenge.

## Results

### **T**he life story of Maria

#### Maria’s background

Maria grew up as the eldest of five siblings in a town on the western coast of Norway. Her mother was a homemaker. Her father had a university degree and a good job. Both of her parents came from a working-class background. Maria gave few specifics about her childhood, except to say “*Through all the years my parents always stressed that we had to get an education. That’s what I was weaned on.”* She had no clear or specific memory of **why** she felt that her parents gave such high priority to education. She began a programme to become an auxiliary nurse because her parents strongly suggested it. The way she describes it, Maria passively accepted what her parents had proposed. However, Maria did not enjoy the role of an auxiliary nurse “*because auxiliary nurses are at the lowest rung of the ladder*”. To explain what she meant, she described an experience in which she was in the on-call room together with a younger nurse who referred to her as “*a ‘witty’ auxiliary nurse. She actually meant it in a good way, but I felt a huge difference in rank since I was only mentioned as the auxiliary nurse who was witty*.” Maria said it was “*degrading*” and “*humiliating*”. She also recalled how the doctors and nurses discussed matters in the on-call room, *“…and I thought that I could have contributed some valuable information and such, but there was really no room for me to participate, and I also felt a little…uh…it was a feeling of being pushed aside…I didn’t feel at home in that role, on the bottom rung without anything to say*”.

As a 30-year-old, Maria began her nursing studies. By then she was married and had a daughter. She described her decision to begin studying nursing as a coincidence, steered by others. She had applied for admission to an art school, but she had some friends who started nursing studies and so she thought, “*Why not*?” Maria said, “*At least I was happier as a full-fledged nurse than as an auxiliary nurse. But I don’t think of being a nurse as a job with high status. (…) Uh…of course such things are also important in our lives. How we see ourselves. It would be fun to introduce myself as a doctor once in a while.*”

### Maria’s experiences as a nurse

After completing her nursing degree, Maria had another daughter and worked in part-time positions for periods of one to two years in various hospital units. In the past 10 years she has worked in the cancer unit in an 80 percent position, and she still works there. Maria clearly remembers her first patients there, including: “*The elderly artist – yes, I remember him very well. He talked and told me about his life, and I sat and listened, and it was very, very, very, it was sort of like I was high when I left his room because it was so fantastic. Uh, but it’s a little strange sometimes. The first time I meet people, it’s so wonderful, you know, but it doesn’t always stay that way (…) I’m not so good at following up (…) Maybe I gave the impression, maybe he had a higher opinion of me as a starting point – that I understood more, was more capable, was smarter, that I gave that impression, but then there wasn’t so much to me after all (…) that I wasn’t so exciting after all.”*

Maria gave several examples of this: “*I remember another patient. She was a lovely woman with a high level of education and a great job and such here in the city. Very pleasant and very poised and polite, (…) and she talked about all kinds of things, (…), but after a while it became, uh, embarrassing. (…) I don’t know if I end up in a sort of a servile role after some time, where I’m so incredibly nice, uh, ‘Do you want something to eat?’, ‘Do you want something to read?’, ‘What can I do for you?’, ‘How are you feeling?’, ‘What is wrong?’ and such. Something about that is not right! Maybe (*she laughs a little*), it’s like I’m glad to be finished with them, in a way.*”

Maria said that even though she experienced many sad situations in the cancer unit, she enjoyed her work very much. “*I’m often glad when I leave work*,” she said. She told about an incident in which she had been praised by a patient and had told the story at the dinner table at home: *“…so my daughter says ‘I also want to be a nurse!’ (*she shouts*). No…uh…it’s not that I need so much praise, but the****gratitude****you encounter – that feels wonderful! Uh..that is…the feeling that you****can****help, that it****was****important in a small way that I was there at that precise time and had time to talk with him or her. That feels good*.”

In spite of this, Maria said she would not advise her daughters to become nurses. This is not because of what the job entails, but it is related to “*the experience of inferiority. I as a nurse feel small – that I don’t have a position*.” She also said: “*It has to do with status and salary and the like. That is also significant in our lives*.” Maria reflected over the fact that it was perhaps the satisfaction of working in the cancer unit that made her feel she enjoyed her work.

### How the experience has changed Maria

Maria told about the individuals she met and how “*incredibly well the patients handle news about death and suffering and sorrow”.* Then she reflected*: “…but whether you become so much wiser, you wish that you could sometimes pour out your experience (*she laughs a little while she talks*) and really be supportive and helpful. I often feel small when I encounter those people. You don’t exactly know what to say*.” Then she said: “*I don’t know if I do my job any better today than I did 10 years ago*.” She mentions practical things she has become more confident about, but she does not know if she has gotten any better at being a “*conversation partner*”. Both privately (as a woman) and on the job (as a nurse), it seems that Maria experienced “*the conversation*” as a demand to listen without getting anything in return. “*Sometimes in social situations you can get the feeling that it’s the nurse sitting there and I’m present for the sake of others.*”

Towards the end of the interview, Maria said that she had given some thought to what she believed I wanted to ask her based on the information material for the interview: *how my choice of profession has affected me as a person*. Maria hesitated and said that “*Personally I think genetics and such are stronger. I don’t think people change very much. Uh…I think I resemble my father, and I think he’s similar to me too (*she laughs when she talks*), and he has not even been close to this profession*.” She said that he is a good listener. Maria did not talk about her mother at all in the interview.

Maria also said that she does not feel proud of her identity as a nurse. “*I don’t feel proud at all really*” and she thought maybe this is because “*nursing is so diffuse. Anyone could do it. Being a fellow human being, or being supportive and helpful, is not something that requires…so much*”. She laughed softly and said “*I shouldn’t have said that*”.

Although Maria said that she was not proud of her identity as a nurse, she did say that she thinks the nurses in the unit where she currently works “*have the best attitudes I have ever encountered. If I or any or my loved ones were to get sick, I would feel safe and secure with each and every nurse here.*” However, Maria criticized the fact that the nursing profession is “*dominated by women*” and said “*working with so many women can get a little boring*”. But then she elaborated: “*I think women are fantastic*.” She said she liked *“…strong, tough, admirable women who dare to speak their minds, who stand up for what they believe and take up space*.” But she thinks it was difficult to be around women who only were “*good and kind and mostly caregiving types.*” After pausing, she said: “*It happens a lot that people sit in the on-call room and groan and complain about the sorrow and misery we encounter on a daily basis and dream about what it would be like to work in a flower shop or have a totally different job....*”.

Maria concluded her story by saying that she “*has thought many times that other things would have been exciting*”. Although she found her daily encounter with people with problems to be challenging, she did not think that there were any changes in the job itself. “*The things that change are the different people and new forms of treatment, but there are not big,****new****things to learn otherwise, or challenges otherwise, in any way*.” And if she could start over, she thinks she would have taken more education – if she had “*had the energy*”.

When she finished her story, she said that she felt she had revealed too much about her private life. And then she said, “*I sit here and think, did I seem a little stupid, did I seem trite and simple*?”

## Discussion

### To denigrate a competence that she at the same time wants to achieve

Maria's concrete experiences on becoming an adult, and encountering colleagues, patients, family and friends, reveal her concern of how little status she has as a nurse. All of us carry our own needs and inner conflicts into the workplace with us, which makes us especially vulnerable to external factors that impact our work [28]. It seems that Maria is particularly vulnerable to the external factor that status plays in her life. Through the analyses of her story, a dilemma connected to her perception of her role and herself comes to the surface. We learn that Maria denigrates the content in the nurse’s work by saying that it is something that “anyone” can do, and that care and conversation with the patients is not a proper “competence”, since it does not require any specific knowledge and is nothing to be proud of. At the same time, she says it is care and conversation that are the most difficult to be able to provide to patients over time. In other words, she put herself in an impossible situation. She can attempt to be good at something that anyone can do (and therefore nothing to be proud of), or she could give up and try to be better at it and thus get the feeling that she is not capable of something that “anyone can do”. The concern about her dilemma is that it prohibits her in her professional development.

In the following, I will discuss Maria’s self-understanding configured by the dilemma. First, I try to understand her self-understanding in a social historical context. Second, I interpret what might be at stake in a psychoanalytic perspective (a psychic interpretation). I next turn from the inner experience of Maria to the outer (a social interpretation). Here, I relate to relevant nursing literature, confirming and discussing Maria’s experience of the nursing role as being repressive. In the last section in the discussion (a psycho-social approach to professional development), I discuss possible ways for Maria to escape her dilemma, which implies that her self-understanding must be targeted psycho-socially, both by looking inside (for anxieties and defences) and outside in trying to formulate definitions of professional care.

### Maria’s social historical context: experiences she carries into her professional life

In her interview, Maria told very little about her background and childhood. However, some clues were available to the panel, to the research group and to me, thereby enabling us to create a hypothesis about her childhood and how it might have influenced her life. The structure in Maria’s biography resonates with the historical context of many girls who grew up in Norway in the 1960-1970s. During the time of Maria’s childhood, the father was an important authority figure in most families. Maria’s father had broken out of the working class background he came from, and with his education lifted both himself and his family into another category, namely that of the middle class. A hypothesis from this is that Maria acquired a “responsibility” from home (without a direct expression) to represent the new generation in their family, and of those who abandoned the working class and acquired a new capital of positions, money and intellectual capacity.

In Maria’s youth during the 1970s, the women’s movement grew tremendously and gender roles were an important theme in the societal debate. As a daughter in a family, Maria may have possibly felt ambivalence towards her mother, who was only a housewife. Could this have had consequences for Maria’s relationship to caring and what she perceived as being feminine? Another feature with the 1970s in Norway was the “education explosion” [29] (p. 318), which was a sign of an upward swing in social mobility. The 1970s were also characterized by political engagement, as it was usual to demonstrate, with people taking part in protests in the streets and proclaiming their rights. Even so, the trend turned towards the end of the 1970s. A lot of money circulated in Norway because of the oil industry, and in the 1980s when Maria was a young adult struggling to decide what to do with her life, we could see big cabin cruisers, expensive cars and luxury cabins, all of which demonstrated to the entire world that to succeed economically also meant to succeed socially.

As a result, Maria grew up with ideals from several worlds, including ideals about education, self-realization, money and success. These ideals stood in contrast to the role she experienced in reality: a servile “servant” role and the “kind and nice” listening nurse, both at work and in her private life. These contrasts helped to shed light on Maria’s statements about her not identifying in a positive way with the role of a nurse, even after a long working life.

### A psychic interpretation

In a psychoanalytic perspective, an infant is exposed to both conscious and unconscious adult expectations. The infant absorbs and incorporates aspects of the parental unconscious, including their wishes, expectations and hopes for this child [30] (p. 544). The child develops a sense of agency – a belief in having the capacity to bring about the desired state. An interpretation of Maria’s strong feelings of denigration and hope for an improved status and acknowledgement can be that she is unconsciously haunted by her parents, haunted by “ghostly” emotions [31]. On a conscious level, Maria has a strong feeling that education is important, as she was “*weaned on it*”. On an unconscious level, the mental states from parents are ascribed. Nevertheless, Maria can get the uncomfortable feeling of not being a representative for the new generation in her family.

People unconsciously use defences in order to keep from having to deal with their own uncomfortable feelings. Maria thinks she is good at the beginning of the relationship with her patients and the conversation between the two. She is a good listener (a trait she ascribes to her father), and she feels capable when it comes to informing patients about the procedures and routines in their treatment, but she explains that she thinks it is difficult to continue a good relationship with her patients. She says that she is afraid that the patient will discover that she is not that clever after all, and that “*there wasn’t so much to me after all*”. If we understand this as a projection, it may be Maria herself who is afraid to discover that she “*isn’t so exciting after all*”.

At the same time, it can be interpreted as a defence against the experience of falling short when she says that care and conversation are not about proper competence, but something that “*anybody can do*”. Maria has grown up with a sort of need to achieve a certain status, and feels that her identity is threatened by the experience that care has none. She does not fight for it, but instead denigrates caring competence. In a psychoanalytical frame, this could be identified as identification with an aggressor. A simple explanation for this is that if you cannot beat them, join them. These defences of projection and identification with an aggressor that I have pointed to in this section helped Maria distance herself from a feeling of failure in some professional relationships. On the other hand, these defences also prohibit her from further development in professional relationships.

### A social interpretation

Let us turn from Maria’s inner to outer experience to better understand her dilemma. Maria is not alone in experiencing the nursing role as being repressive. Nursing literature confirms that nurses feel a lack of recognition [6], and that nurses themselves also find it difficult to present a meaningfully distinct role [32]. McCrae *et al*. [32] argue that nursing contributes to its undervalued status by creating job titles such as “advanced nurse practitioner” (p. 771). Sturegon [33] suggests a possible reason for nurses’ low status in that there is an “over-emphasizing” of the role of the nurse in terms of interpersonal relationships and emotional engagement. However, this is a statement much nursing research will argue against [5,13,34-38]. According to Reverby (1987) [5], nursing’s central dilemma is “being ordered to care in a society that refuses to value caring” (p. 131). Does this societal refusal of the value of caring have something to do with nursing being a woman’s work? Henderson [5] says that nursing is invisible from being a woman. Even if women today claim the right and need to seek autonomy and personal gratification beyond caring commitments, a feminine maternal ideal underpins women’s practice of their work relationships [39,40]. The expectations remain influenced by a stereotypical understanding of nursing (as kind, empathetic and caring), an image that remains prevalent in society [40]. Some will argue that this expectation is positive for women in their professional work relationships, something that makes the identification with their work easier because the role is close to their gender identity [41]. In a previous article [39] we argued against the latter, suggesting instead that female relationship workers have a constrained portrait of themselves, leaving little opportunity and permission to explore the difficult emotional and situational complexities that they experience in their professional practice. In the case of Maria, we receive support for this argument. Maria wants to divorce herself from the gendered constraints, as she does not want to identify with the feminine ideal. Instead of nurses who are “*kind and nice*”, she wants to identify with “*strong, tough admirable women”*. In other words, the societal disparagement of caring work, together with the female mothering ideal connected to nursing, contributes to Maria’s struggle to identify with the caring part of the nursing role. At the same time, this is an obstacle for her development in professional relationships.

### A psycho-social approach to professional development

We have now seen how Maria’s self-understanding is formed by her inner and outer past, as well as by present experiences and expectations. Maria’s experience of falling short over time in her relationships with patients, that she is not good enough, is not a result of feedback from patients or colleagues, but rather from her own self-understanding. This self-understanding prohibits Maria from further development in her professional relationships as she is locked in the dilemma described earlier: She can try to be good at something everybody can do (and therefore nothing to be proud of) or she can give up on trying to be better at it, and thus get the feeling that she is not capable of something “*anyone can do*”.

Still, there are ways to escape this dilemma, which implies that Maria’s self-understanding must be targeted. Let us have another look at Maria’s experience using a different set of glasses – different from her own self-understanding.

According to Arbon [11], the way Maria is able to focus on the caring, interpersonal and connecting side of practice in her story is a sign that she is an experienced professional nurse. Being troubled by aspects of the care situation is related to several other features that develop with experience, he claims. In accordance with this, Zolnierek [42] found there was a connection between “knowing the patient” and expert practice, while Henderson [5] calls the use of emotions in nursing a “high-level skill” (p. 135). Establishing relationships is to encounter vulnerability, both within oneself and within others, which calls forth every aspect of the professional’s self. Maria should be able to accept herself as vulnerable and “good enough”, and not perfect. However, Maria’s view of herself is in line with other nurses who are critical of their own performance, feeling that it falls short of accepted professional standards when they show their own vulnerability in patient care [43]. Yet, Maria does not always denigrate the caring role, sometimes describing how happy she is when she has the experience of being able to give good care, and how her daughter shouts: “*I also want to be a nurse!”* Her ambivalence is obvious (she is proud, but also shameful of being proud).

Vulnerability and ambivalence connected to relationships make the experiences in the relationships difficult to access for learning [14]. Hence, stagnation can be the result.

Maybe it is the experience of stagnation that makes Maria say that she feels a need for challenges, even if she encounters new patients who fight for their lives every day. Maria imagines that the possibility for change and development means starting with something else in another place. Maria is not alone with her feelings of wanting to go away and receive the opportunity to do something completely different, as we also hear how many in the on-call room have dreams of going away.

Nonetheless, the possibilities for change and development can be found in the nurses’ own practices. In that case, the relationship with the patient, striving to “knowing the patient” [42] has to be considered as a “high-level skill” [5], as well as something that the nurses feel challenged by and are challenged to develop. This will need a reflective practice through an open dialog and supervision that can help enhance a self-awareness of the inner conflicts related to ongoing distressing professional vulnerabilities. The individual nurse and the group of colleagues can understand and be aware of their own and collective anxieties and defences, in addition to being capable of formulating realistic definitions of professional care [28,37,39,43-45]. The challenge for Maria, and for other nurses and policymakers, is to facilitate and value the development of the intangible emotional and empathic qualities of nursing practice [11]. In my own study of experiences from a Norwegian hospital [3], it appears to be a problem that the nurses and the hospital play down or turn a blind eye to the experience of anxiety and vulnerability in their relationships. This seems increasingly more problematic, since nursing is progressively more determined as an economically driven management model of service delivery [4].

Professional identity is likely to be a major factor in the satisfaction and retention of nurses [32,46], which helps to support the significance of nurses becoming conscious about their self-understanding, and how this influences their professional work.

## Conclusion

The biographical interpretive method used in this project makes accessible the inner, somewhat hidden dynamics in the self-understanding of a middle-aged female nurse, which has the capacity to explore the interaction between personal and social factors in shaping her professional practice. The telling of her life story makes it possible to trace a governing idea through her experiences, the experience of being “only a nurse”. The interview process and analysis of her narrative structure reveals hidden depths – Maria’s underlying distress and fears of not being good enough, the suppression of which must consume a considerable amount of energy, and her reserves of reflexivity that largely remain untapped in the context of her work. It suggests that while there may well be biographical reasons for such low self-esteem and self-denigration, the nurse experienced that society at large also actively disparaged the competence of nurses, and in particular the needed relational competence.

Through a careful consideration of this one case, my aim was to provide insight that health professionals can relate to in their own subjective way. This insight may lead to a desire to understand oneself as a part of a professional development. However, if I should attempt to suggest implications for practice and nurse education from this study, I would highlight the importance of exploring vulnerability in relationships, based on the assumption that their vulnerability is both personally and socially constituted. The link between the professional and the personal implies that there cannot be any real professional development without personal development.

## Abbreviations

BNIM: Biographic Narrative Interpretive Method
SQUIN: Single Question aimed at Inducing Narrative
PIN: Particular Incident Narrative
